# Multimodality Imaging in the Assessment of Thoraco-Omphalopagus Conjoined Twin: Lessons to Learn

**DOI:** 10.1155/2012/564036

**Published:** 2012-06-27

**Authors:** Kanaga Kumari Chelliah, M. Z. Faizah, A. A. Dayang, A. A. Bilkis, I. Shareena, M. Mazli

**Affiliations:** ^1^Faculty of Health Sciences, Universiti Kebangsaan Malaysia, Jalan Raja Muda Abdul Aziz, 53000 Kuala Lumpur, Malaysia; ^2^Department of Radiology, Universiti Kebangsaan Malaysia Medical Centre, Bandar Tun Razak, Cheras, 56000 Kuala Lumpur, Malaysia; ^3^Pediatric Surgical Unit, Department of Surgery, Universiti Kebangsaan Malaysia Medical Centre, Bandar Tun Razak, Cheras, 56000 Kuala Lumpur, Malaysia; ^4^Department of Pediatrics, Universiti Kebangsaan Malaysia Medical Centre, Bandar Tun Razak, Cheras, 56000 Kuala Lumpur, Malaysia

## Abstract

Conjoined twins are rare and present a unique challenge to pediatric surgeons and radiologists. An imaging strategy to accurately define anatomic fusion, vascular anomalies, and other associated abnormalities is important for surgical planning and prognostic information. A conjoined female twin with a combined weight of 2.8 kg was born by emergency caeserean. Hence, a computed tomography scan of the thorax and entire abdomen at 1.25 mm slice thickness was performed to delineate the internal structures of the twins. CT-angiography defined specific vascular supply which determined the distribution of shared structures between the twins. An echocardiogram showed four heart chambers with atrioventricular septal defect. To further evaluate the heart chambers, the twin was planned for gated cardiac magnetic resonance imaging. Unfortunately, they succumbed 6 hours apart due to complication of septicemia. Magnetic resonance imaging and CT scan provide excellent anatomic detail, demonstrating organ position, shared viscera, and limited vascular anatomy, whilst angiography defined specific vascular supply, useful in determining the distribution of shared structures between the twins in planning for surgery.

## 1. Introduction

The prevalence of conjoined twins is reported to be approximately 1 in 250,000 live births [1, 2]. Approximately, 40 to 60% of conjoined twins are stillborn, and about 35 percent survive only one day. The overall survival rate for conjoined twins ranges between 5–25%. Conjoined twins are genetically identical and are of the same sex. They develop from the same fertilized egg, and they share the same amniotic cavity and placenta. There are nearly a dozen different types of conjoined twins, 40% and 35% are thoracopagus and omphalopagus, respectively [1, 2]. Such twins are classified according to the most prominent site of connection: the thoracopagus (thorax), omphalopagus (abdomen), pygopagus (sacrum), ischiopagus (pelvis), craniopagus (skull), cephalopagus (face), or rachipagus (back). Planning of surgical separation requires prior accurate preoperative imaging. Computed tomography (CT) and magnetic resonance imaging (MRI) provide excellent anatomic detail demonstrating organ position, shared viscera, and limited vascular anatomy [1]. This paper discusses the application of imaging modalities in detecting anomalies in a conjoined twin.

## 2. Case Report

A conjoined twin of female sex and a combined weight of 2.8 kg was delivered by emergency caesarean section at 33-week gestation. No maternal complications were shown during prenatal checkup. However, no embryogenesis or genetic analysis was done during the prenatal checkup.

The twins had cleft lips oriented face to face and were externally fused from the lower chest to the upper abdomen in the ventral aspect. An echocardiogram showed four heart chambers with atrioventricular septal defect (AVSD).

**Figure 1:**
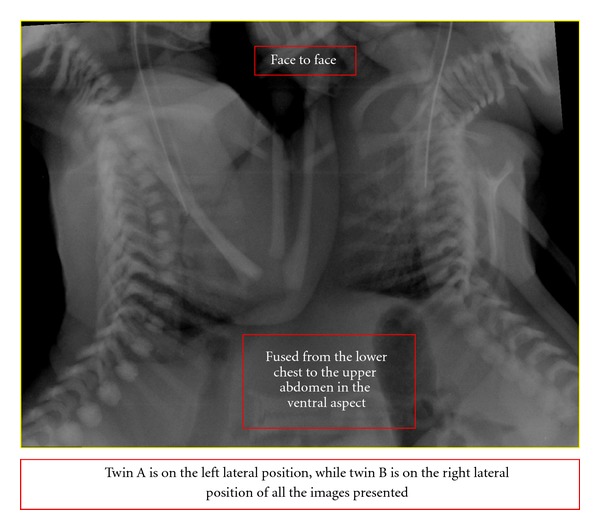
Twin A is on the left lateral position, while twin B is on the right lateral position.

**Figure 2:**
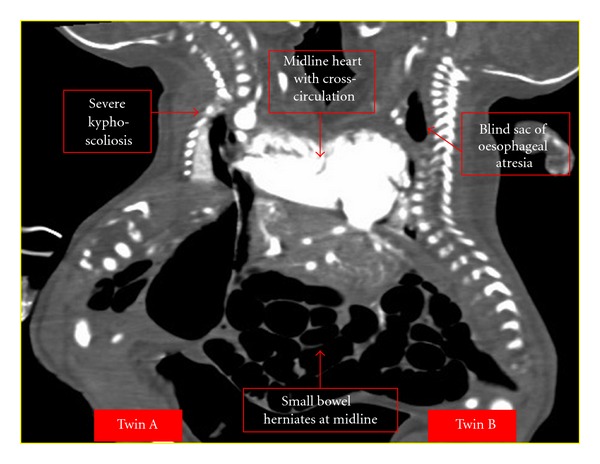
Virtual Reality reconstruction of twins A and B.

 To delineate the internal structures of the twins, a Siemens Somatom 64-slice multidetector CT scanner (Siemens AG, Germany) was used to scan the thorax and entire abdomen at 1.25 mm slice thickness. The anatomical side of the twins was labeled to make sure that there was no confusion as the twins were placed beside each other. Plain scans followed by intravenous injection of 2 mL/kg of Iopamidol 370 (Bracco, Milan, Italy) on twin A was performed to obtain the arterial- and venous-phase images. Twin A was on the left lateral position, while twin B was on the right lateral position (Figure 1). Images were reconstructed using virtual reality imaging as shown in Figure 2. There was a common pericardial sac in both twins, complete cross-heart circulation from twin A to B. On CT angiogram, there was visualization of 6 heart chambers seen on CT angiogram. There was evidence of a large AVSD as seen on echocardiography. The great blood vessels, namely, aorta, pulmonary trunk, and inferior vena cava are right-sided and enter the right atrium in twin A and left-sided in twin B. Both lungs are well developed but show consolidation during repeat CT. A hypoplastic left lung fused liver was seen on the ventral aspect with the presence of cross-circulation.

There was evidence of a distal tracheo-oesophageal fistula, and both twins showed separate portal and hepatic venous system. Other organs in the abdomen like the gallbladder, biliary system, pancreas, and spleen were all separate for both twins. Herniation of upper small bowel was seen into twin B cavity. Two kidneys enhanced normally for both twins A and B. Both twins showed evidence of severe thoracolumbar scoliosis.

**Figure 3:**
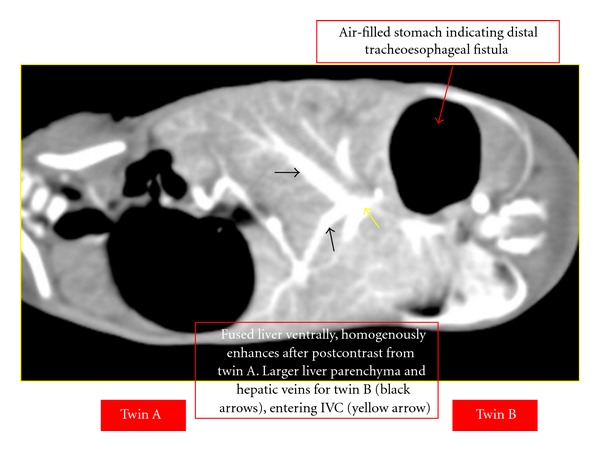
Contrast-enhanced image of abdomen.

**Figure 4:**
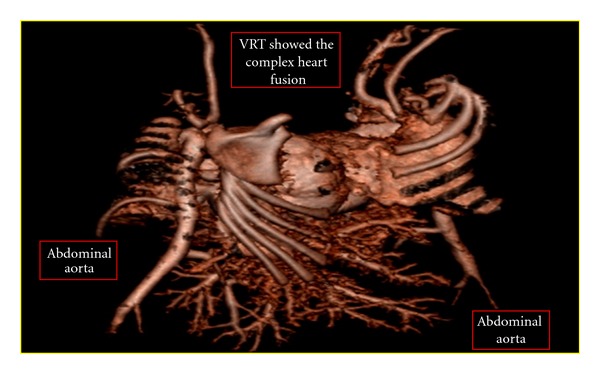
3D image of the cardiac fusion in twins A and B.

 Six days later, a CT angiography was performed with intravenous contrast medium injection into twin B to evaluate the blood supply, the images are shown in Figure 3. A 3D reconstruction to demonstrate the cardiac region was done as shown in Figure 4. The twins were planned for gated cardiac MRI for further evaluation of the heart chambers. Unfortunately, the twins succumbed due to the complications of septicemia because of which the MRI was not performed.

## 3. Discussion

Thoracopagus twins are united face to face from the upper thorax to the umbilicus with a common sternum, diaphragm, and upper abdominal wall. Ninety percent of such twins have a common pericardial sac, and there is always a degree of cardiac fusion in 75% of cases which precludes successful surgical separation [3, 4].

Associated cardiovascular abnormalities are found in 75% of thoracopagus twins with conjoined hearts. These abnormalities vary from a common pericardial sac to atrial and ventricular fusion with or without fusion of other organs. The most frequent atrial malformation in thoracopagus twins is a common atrium with a large atrial septal defect. The most common ventricular malformation is a single ventricle with an infundibular outlet chamber and a large ventricular septal defect. The great arteries are usually not fused, but they are often transposed.

Echocardiography should be used as the initial postnatal investigation to establish the degree of cardiac conjunction and associated structural heart abnormalities. MRI and CT provide excellent anatomic and bone detail, demonstrating organ position, shared viscera, and limited vascular anatomy. Contrast-enhanced imaging allows evaluation of the gastrointestinal and urogenital tracts, and a shared liver requires assessment of anatomy, vascularization, and biliary drainage. Angiography defines specific vascular supply, which is useful in determining the distribution of shared structures between the twins at surgery. Each set of conjoined twins is unique. An imaging strategy to accurately define anatomic fusion, vascular anomalies, and other associated abnormalities are important for surgical planning and prognostic information.

## 4. Conclusion

Conjoined twins are rare and a unique challenge to pediatric surgeons; therefore, multimodality imaging is used for surgical planning. Echocardiography is used as an initial postnatal investigation to delineate gross abnormalities. MRI and CT provide excellent anatomic detail, demonstrating organ position, shared viscera, and limited vascular anatomy in the twins. Angiography defines specific vascular supply, which is useful in determining the distribution of shared structures between the twins.
